# Supplementary material for: Multiplexable and Biocomputational Virus Detection by CRISPR-Cas9-Mediated Strand Displacement
Source: Anal Chem. 2023 May 19;95(25):9564–74. doi: 10.1021/acs.analchem.3c01041 (PMC10255568; doi:10.1021/acs.analchem.3c01041)
Supplement: Supplementary file 1 — ac3c01041_si_001.pdf [file ac3c01041_si_001.pdf]

Supporting Information

Multiplexable and biocomputational virus detection by CRISPR-Cas9-mediated strand displacement

Rosa Márquez-Costa[1,#], Roser Montagud-Martínez[1,#], María-Carmen Marqués[1], Eliseo Albert[2], David Navarro[2,3], José-Antonio Daròs[4], Raúl Ruiz[1], and Guillermo Rodrigo[1,*]

[1]Institute for Integrative Systems Biology (I2SysBio), CSIC – University of Valencia, 46980 Paterna, Spain. [2]Microbiology Service, Clinic University Hospital, INCLIVA Biomedical Research Institute, 46010 Valencia, Spain. [3]Department of Microbiology, School of Medicine, University of Valencia, 46010 Valencia, Spain. [4]Instituto de Biología Molecular y Celular de Plantas (IBMCP), CSIC – Universitat Politècnica de València, 46022 Valencia, Spain. [#]Equal contribution to this work. *Correspondence: guillermo.rodrigo@csic.es

Contents

Supplementary Methods ______ S2 - S4
Supplementary Figures ______ S5 - S15

SUPPLEMENTARY METHODS

Note on the design of COLUMBO elements. In this work, we decided to use standard primers, such as the Charité E-Sarbeco primers and the CDC N1 and N2 primers for SARS-CoV-2. The occurrence of a PAM sequence for Cas9 (NGG) is a minimal requirement that can be met in many cases, as it occurs in the E and N gene amplicons. Nothing prevents using other primers. The position of the PAM sequence within the DNA amplicon determines the length of the sgRNA spacer. In principle, spacers of 20-40 nt should be adequate.

The precise sequence of the displaced strand conditions the sequence and secondary structure of the beacon. If it is rich in GC, the beacon can be designed with a shorter stem. The dynamic range of the beacon is uncertain *a priori*, so functional screening could be performed. Furthermore, for the beacon to interact efficiently with the displaced strand, the R-loop needs to be open in the PAM-distal end. Otherwise, the displaced strand has not sufficient freedom.

Note on the application by RPA. In our nucleic acid amplifications by RPA, a shaking of 10 s at 300 rpm was applied every 2 min during the 30 min of the reaction. In principle, shaking is not required for RPA to work. However, in our hands, we found better amplifications with shaking, especially in the case of clinical samples. In some cases, RPA reactions without shaking produced no observable bands when revealed in a gel.

Activity assessment of different Cas9 versions. A suitable dsDNA molecule, whose sequence is GGCTAAAGAGGAAGAGGACATGGTGAATTCGTAACT, was labelled with a fluorophore (FAM, in 5’) and a quencher (Iowa Black FQ, in 3’) in the PAM-distal ends. A suitable sgRNA, whose spacer is GGCUAAAGAGGAAGAGGACA, was used to perform the CRISPR reactions. These were done in 1x TAE buffer pH 8.5 (Invitrogen), 0.05% Tween 20 (Merck), and 12.5 mM $MgCl_2$ (Merck) at a final volume of 20 µL. The sgRNA-Cas9 ribonucleoprotein was added at 100 nM and the labelled dsDNA at 20 nM. Here, Cas9, Cas9n, and dCas9 were used. Reactions were incubated at 37 ºC for 20 min in a thermomixer (Eppendorf).

Single-point mutation detection with CRISPR-Cas9. Different E gene amplicon variants harboring substitution mutations in the PAM or the protospacer (in the seed region, *i.e.*, the

PAM-proximal region) were chemically synthesized (IDT). Wild-type Cas9 and HiFi Cas9 from IDT were used. According to IDT, HiFi Cas9 has similar on-target potency to wild-type Cas9, but with significantly reduced off-target effects, then allowing for precise targeting. CRISPR reactions were performed as already indicated.

Note on coupling the amplification and detection reactions. Because the beacon interacts in the PAM-distal region and the R-loop needs to open at that point, the forward primer (with respect to the PAM) overlaps with the region targeted by the beacon. Therefore, the primer may interfere in the detection reaction. This was solved by purifying the amplified material.

To avoid the purification step, we devised a strategy based on cleaving the resulting DNA amplicon in the PAM-distal region with a restriction enzyme. In this way, the forward primer (with respect to the PAM) and the beacon do not significantly overlap. A particular restriction site can be introduced with the primer or be present in the original sequence. In principle, different restriction enzymes may be used. Here, we used XbaI, which has good efficiency in our reaction buffer. We designed a new primer to amplify the SARS-CoV-2 E gene.

CRISPR-Cas9-based detection with no prior purification. From test samples containing the SARS-CoV-2 E gene, DNA amplicons were generated by PCR as already indicated, this time with 45 cycles. Then, 6 μL of non-purified sample was used in the CRISPR reaction. This was performed in 1x TAE buffer pH 8.5 (Invitrogen), 0.05% Tween 20 (Merck), and 12.5 mM $MgCl_2$ (Merck), with the sgRNA-Cas9 ribonucleoprotein at 100 nM, the molecular beacon at 100 nM, and XbaI at 0.5 U/μL, for a final volume of 20 μL. Reactions were incubated at 37 ºC for 20 min in a thermomixer (Eppendorf).

Note on the biocomputing capability with CRISPR-Cas9. The displaced strand upon CRISPR-Cas9 targeting was used to interact with a complex of hybridizing ssDNA molecules, instead of a molecular beacon. The mechanism of toehold-mediated strand displacement was exploited, that is, an enzyme-free reaction to exchange DNA strands. For that, one of the ssDNA molecules forming that complex was designed to have an overhanging region (*i.e.*, a toehold) to seed the interaction with the displaced strand of the N1 amplicon, and another ssDNA molecule was designed to have a toehold to interact with the displaced strand of the E amplicon. Such overhanging regions were at least 6 nt

long. In this way, the complex of hybridizing ssDNAs was responsive to two input signals. The complex was designed to have a sufficiently high melting temperature to be stable in the absence of inputs; in this case, 35 bp were formed. In the case of the OR gate, one fluorophore and one quencher were used. They were arranged to obtain a fluorescent signal when at least one of the N1 or E amplicons was present in the medium. In the case of the AND gate, one fluorophore and two quenchers were used. They were arranged to obtain a fluorescent signal only when both amplicons were present. Here, we designed AND and OR gates, but nothing prevents designing further logic systems.

SUPPLEMENTARY FIGURES

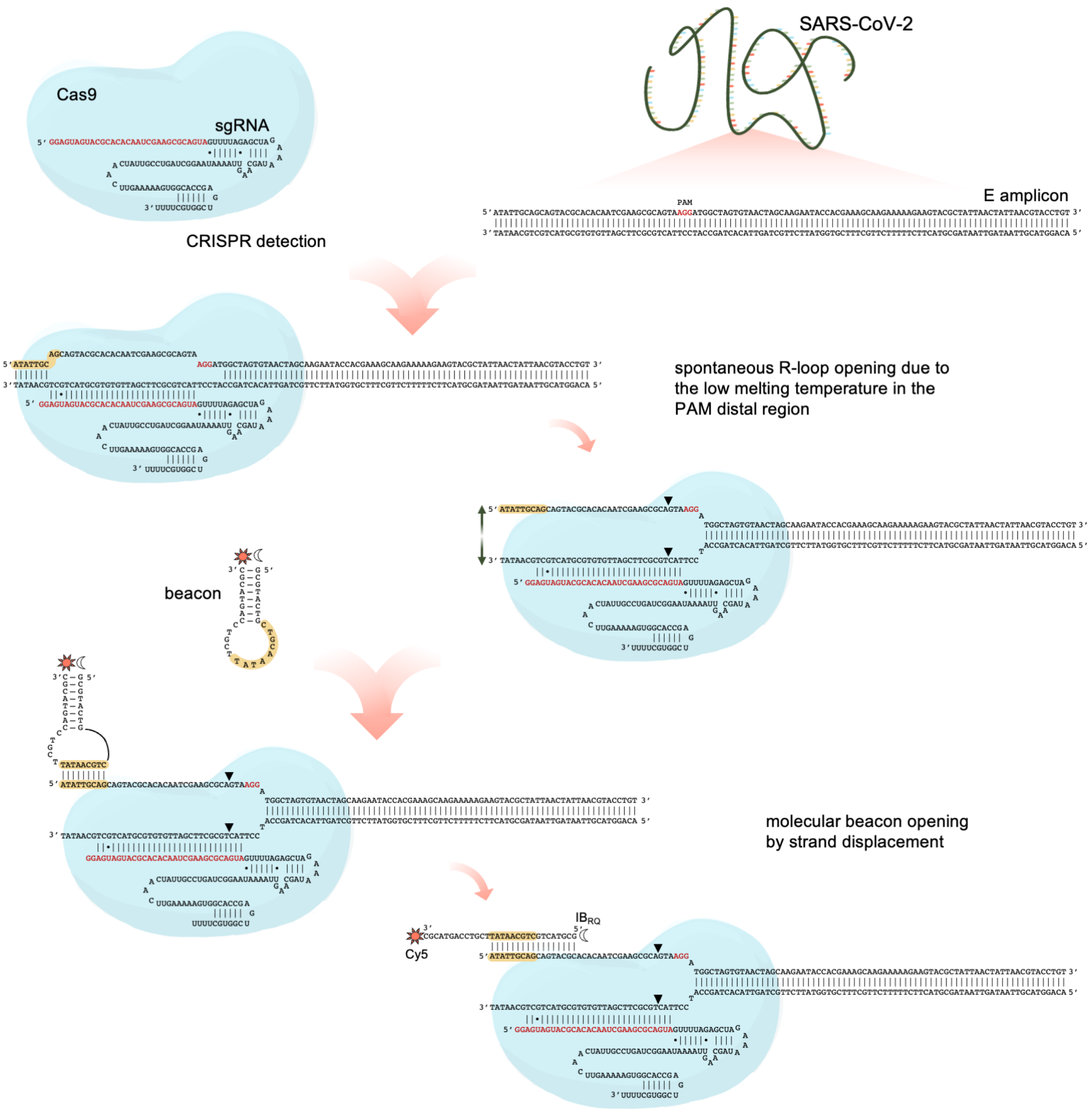

Figure S1: Detailed schematics of nucleic acid detection through CRISPR-Cas9-based strand displacement. In this example, a DNA amplicon from SARS-CoV-2 E gene was generated, containing a PAM (shown in red) for Cas9 recognition. A preassembled CRISPR-Cas9 ribonucleoprotein targeting the amplicon (sgRNA spacer marked in red) could then be used for sequence specific detection. The spacer starts by GG as it is *in vitro* transcribed by the T7 polymerase. The resulting R-loop was open by the PAM-distal region due to a low melting temperature in the DNA end (in this example, 7 base pairs). The displaced strand could interact with a properly designed molecular beacon (seed

regions marked in yellow), which opens afterwards. The molecular beacon was labelled with the fluorophore Cy5 (sun icon) in the 3’ end and the dark quencher Iowa Black RQ (moon icon) in the 5’ end.

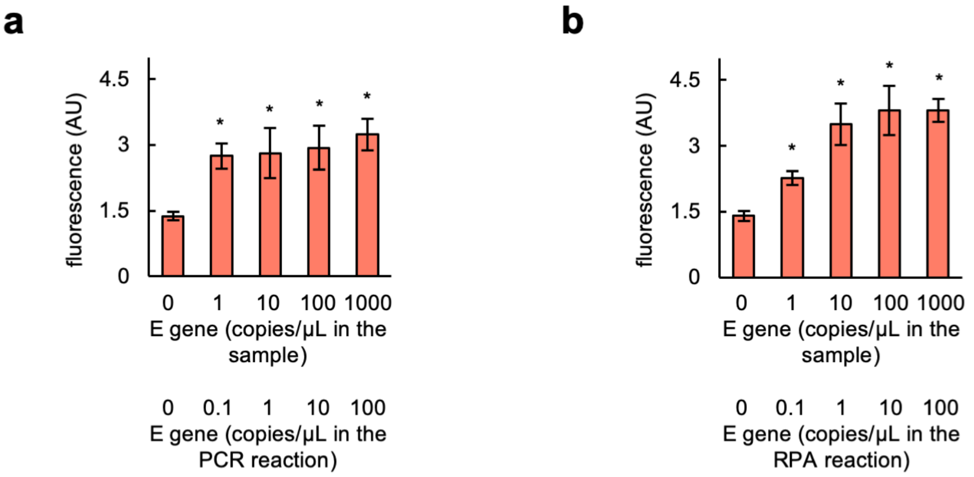

Figure S2: Limit of detection assays. a) Fluorescence-based characterization of the detection with COLUMBO-PCR. b) Fluorescence-based characterization of the detection with COLUMBO-RPA. Test samples containing the SARS-CoV-2 E gene at different concentrations were used. Error bars correspond to standard deviations (*n* = 3). *Statistical significance (Welch’s *t*-test, two-tailed $P < 0.05$).

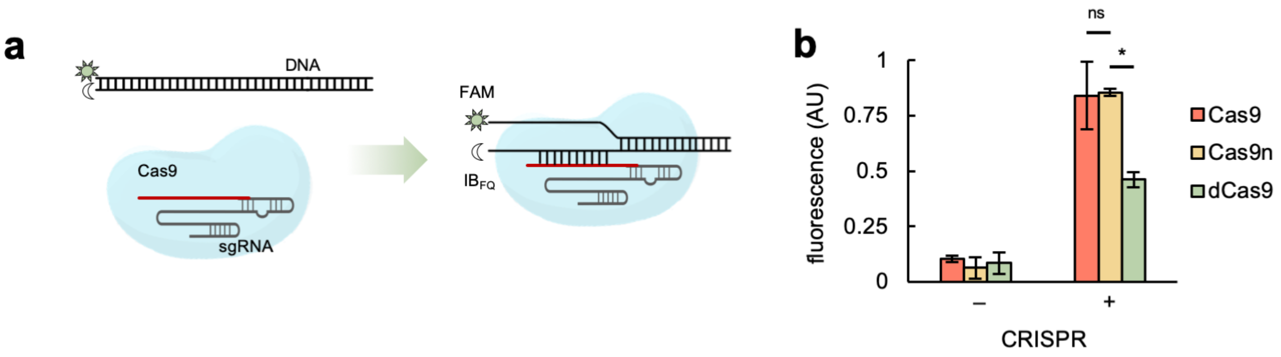

Figure S3: Activity assessment of Cas9 versions. a) Schematics of a test to assess the DNA targeting activity of a CRISPR-Cas9 ribonucleoprotein. The targeted DNA was labelled with a fluorophore (FAM) and quencher (Iowa Black FQ). Upon targeting, the fluorophore is separated from the quencher, then producing a fluorescence signal. b) Fluorescence-based characterization with different Cas9 versions (Cas9, Cas9n, and dCas9). Error bars correspond to standard deviations (*n* = 3). *Statistical significance (Welch’s *t*-test, two-tailed $P < 0.05$). ^{ns}Not statistically significant.

a

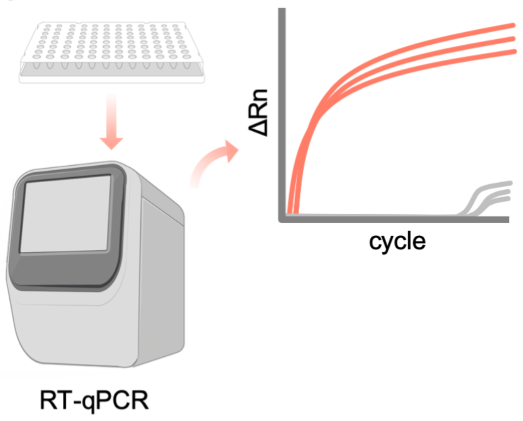

Patient	Sample	Primers	Diagnostic at the hospital	C_T at our lab
P1	nasopharyngeal	N1	Negative	>30
P2	nasopharyngeal	N1	Negative	>30
P3	nasopharyngeal	N1	Negative	>30
P4	nasopharyngeal	N1	Positive	17 - 18
P5	nasopharyngeal	N1	Positive	18 - 19
P6	nasopharyngeal	N1	Positive	17 - 18
P7	nasopharyngeal	N1	Positive	17 - 18
P8	nasopharyngeal	N1	Positive	15 - 16
P9	nasopharyngeal	N1	Positive	20 - 21
P10	nasopharyngeal	N1	Positive	24 - 25
P11	nasopharyngeal	N1	Positive	26 - 27
P12	nasopharyngeal	N1	Positive	23 - 24
P13	nasopharyngeal	N1	Positive	24 - 25
	water	N1		>30

b

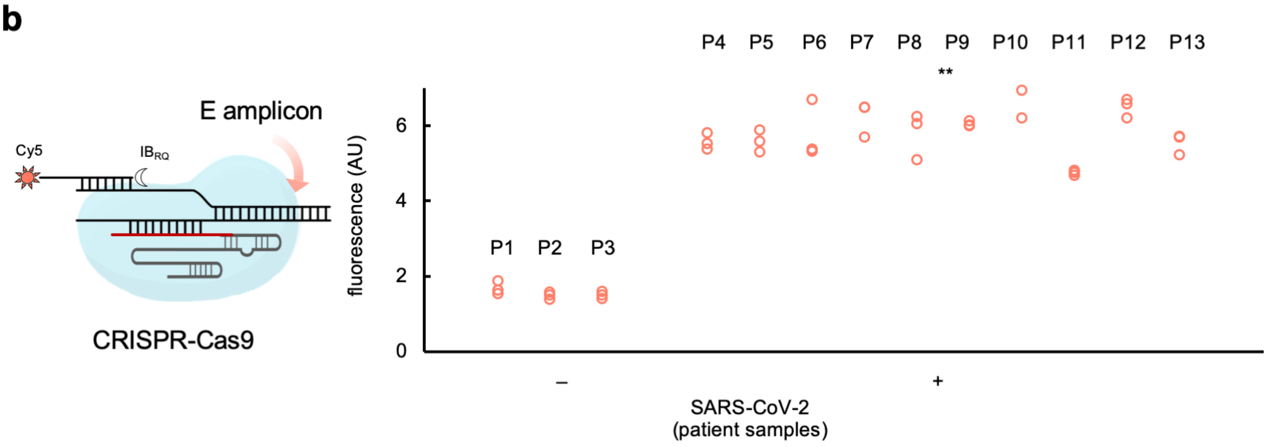

Figure S4: RT-qPCR characterization of patient samples. a) Characteristics of the different patient samples used in this work, including the C_T value (N gene). b) Fluorescence-based characterization of all samples with COLUMBO-PCR (E gene). **Overall statistical significance (Welch's *t*-test, two-tailed $P < 0.001$).

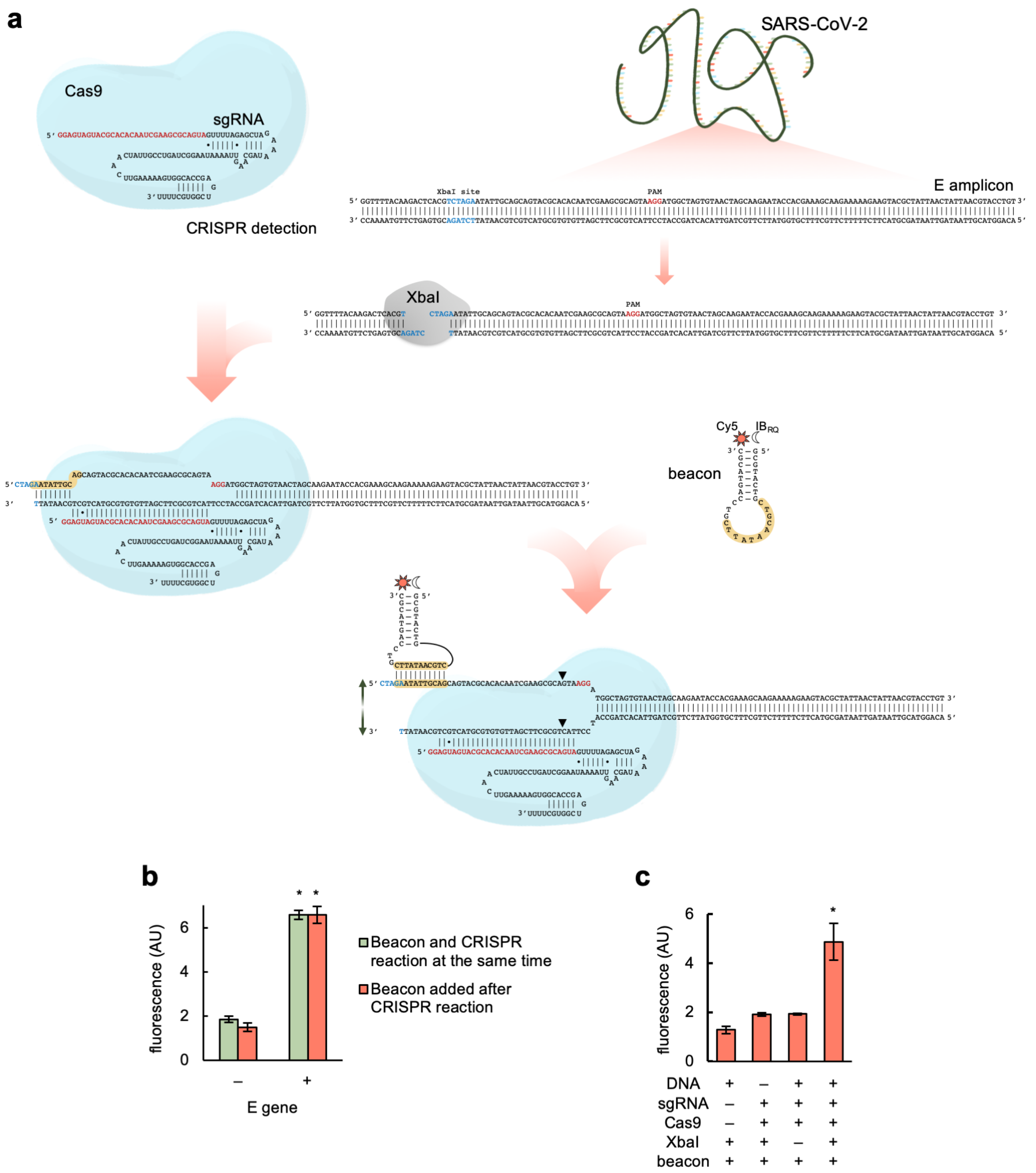

Figure S5: Nucleic acid detection through CRISPR-Cas9-based strand displacement with no prior purification. a) Schematics of the global reaction of amplification and detection of a DNA product from SARS-CoV-2 E gene, containing a PAM (shown in red) for Cas9 recognition and the XbaI restriction site (shown in blue). XbaI was used to cleave the amplicon in the PAM-distal region. A preassembled CRISPR-Cas9 ribonucleoprotein targeting the cleaved amplicon (sgRNA spacer marked in red) was then able to displace a strand so that the molecular beacon could interact with and change its conformation (seed

region for this interaction marked in yellow). The molecular beacon was labelled with the fluorophore Cy5 (sun icon) in the 3' end and a dark quencher (moon icon) in the 5' end. In this way, the purification step was avoided because the primer and the beacon target different regions. b) Fluorescence-based characterization of the detection when the beacon is added at the beginning of the CRISPR reaction. c) Fluorescence-based characterization of the detection with no prior purification, incubating at the same time the CRISPR-Cas9 ribonucleoprotein, the beacon, and the restriction enzyme (XbaI). Error bars correspond to standard deviations (n = 3). *Statistical significance (Welch's t-test, two-tailed $P < 0.05$).

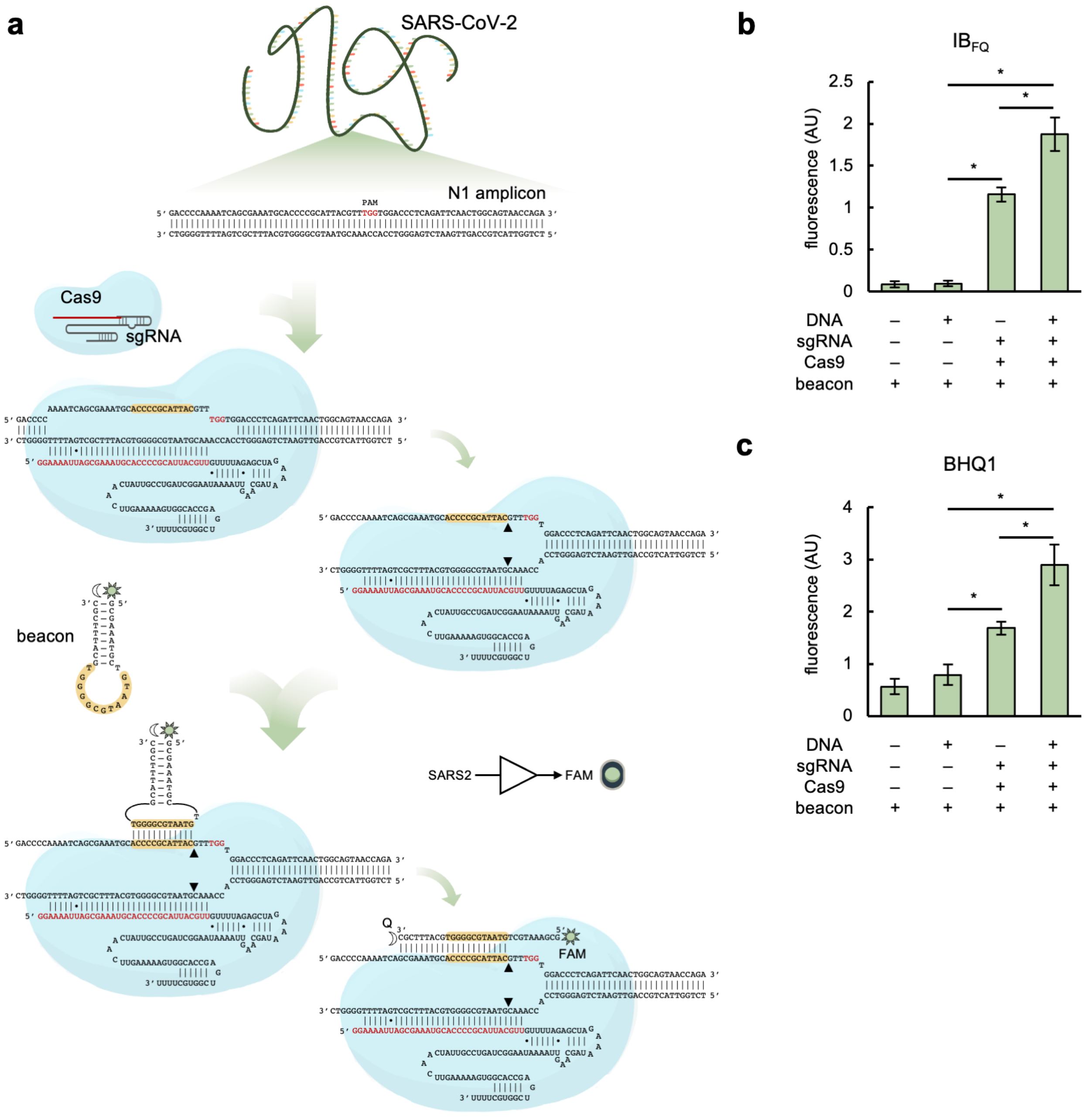

Figure S6: Nucleic acid detection through CRISPR-Cas9-based strand displacement in the PAM-proximal region. a) Schematics of the global reaction of amplification and detection of a DNA product from SARS-CoV-2 N gene (N1 region), containing a PAM (shown in red) for Cas9 recognition. A preassembled CRISPR-Cas9 ribonucleoprotein targeting the amplicon (sgRNA spacer marked in red) was then able to displace a strand so that the molecular beacon could interact with (in the PAM-proximal region) and change its conformation (seed region for this interaction marked in yellow). The molecular beacon was labelled with the fluorophore FAM (sun icon) in the 5' end and a dark quencher (moon icon) in the 3' end. b,c) Fluorescence-based characterization of the detection; amplifications performed by PCR. A high signal was observed in absence of the DNA amplicon and presence of the CRISPR-Cas9 ribonucleoprotein, indicating an unwanted interaction between the sgRNA with the beacon (as in this case the sgRNA spacer contains the seed region). In b) the quencher IB_{FQ} was used, while in c) the quencher BHQ1 was used. Error bars correspond to standard deviations (n = 3). *Statistical significance (Welch's t-test, two-tailed $P < 0.05$).

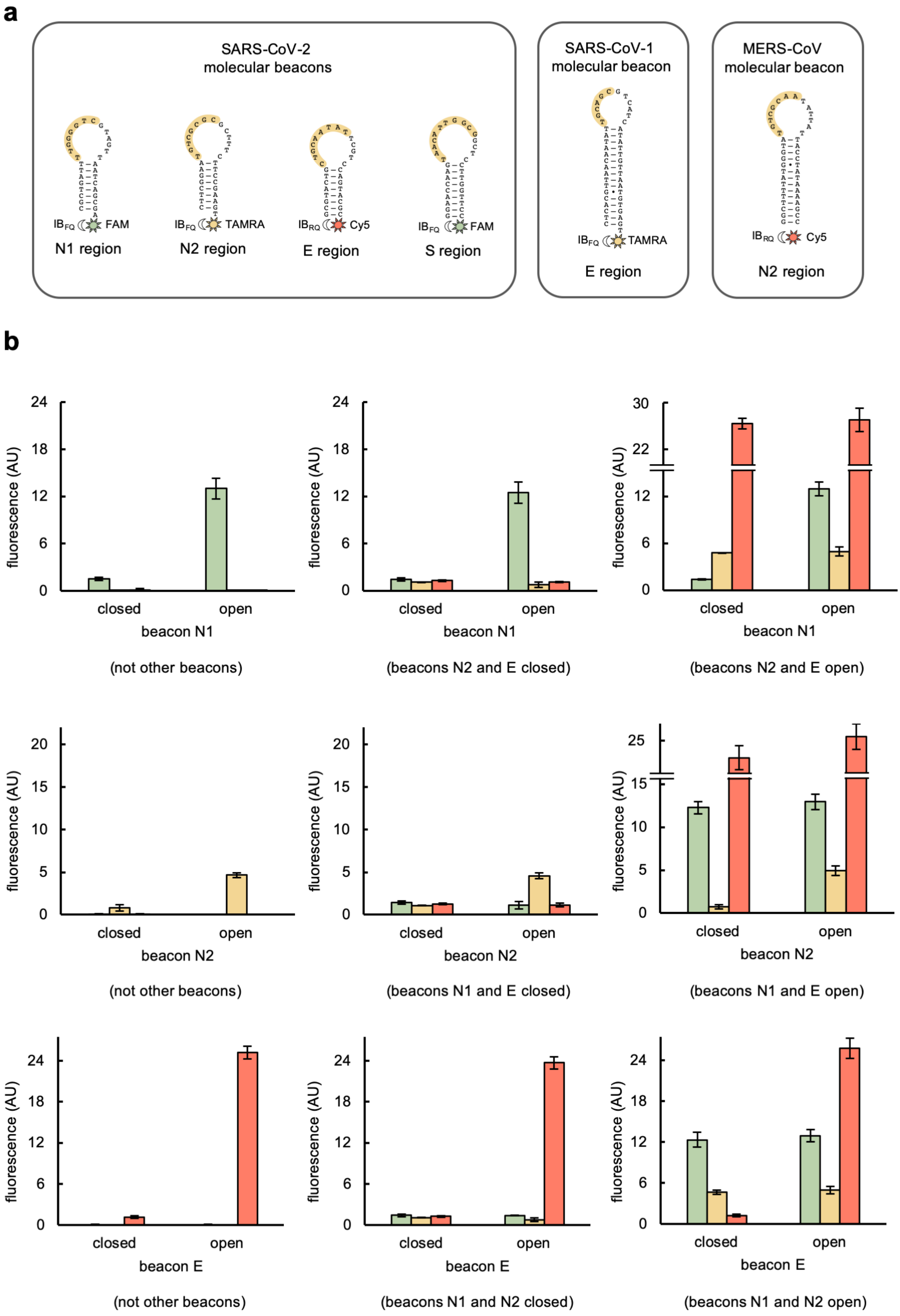

a
SARS-CoV-2
molecular beacons
IBFQ FAM
N1 region
IBFQ TAMRA
N2 region
IBRQ Cy5
E region
IBFQ FAM
S region
SARS-CoV-1
molecular beacon
IBFQ TAMRA
E region
MERS-CoV
molecular beacon
IBRQ Cy5
N2 region
b
fluorescence (AU)
closed
open
beacon N1
(not other beacons)
(beacons N2 and E closed)
(beacons N2 and E open)
beacon N2
(not other beacons)
(beacons N1 and E closed)
(beacons N1 and E open)
beacon E
(not other beacons)
(beacons N1 and N2 closed)
(beacons N1 and N2 open)

Figure S7: Structure and performance of the molecular beacons. a) Secondary structures of the designed molecular beacons (stem-loop folding) to detect SARS-CoV-2, SARS-CoV-1, and MERS-CoV, labelled with the corresponding fluorophores (sun icons; FAM, TAMRA, or Cy5) in the 3' end and dark quenchers (moon icons; IB_{FQ} or IB_{RQ}) in the 5' end. The seed region to interact with the displaced strand is marked in yellow. b) Fluorescence-based characterization of the performance of the molecular beacons to detect SARS-CoV-2 when they work alone or in the presence of other beacons. Open beacons obtained by hybridization with appropriate oligonucleotides. Error bars correspond to standard deviations (n = 3). Effect of other beacons not statistically significant (one-way ANOVA test, independent samples, P > 0.05).

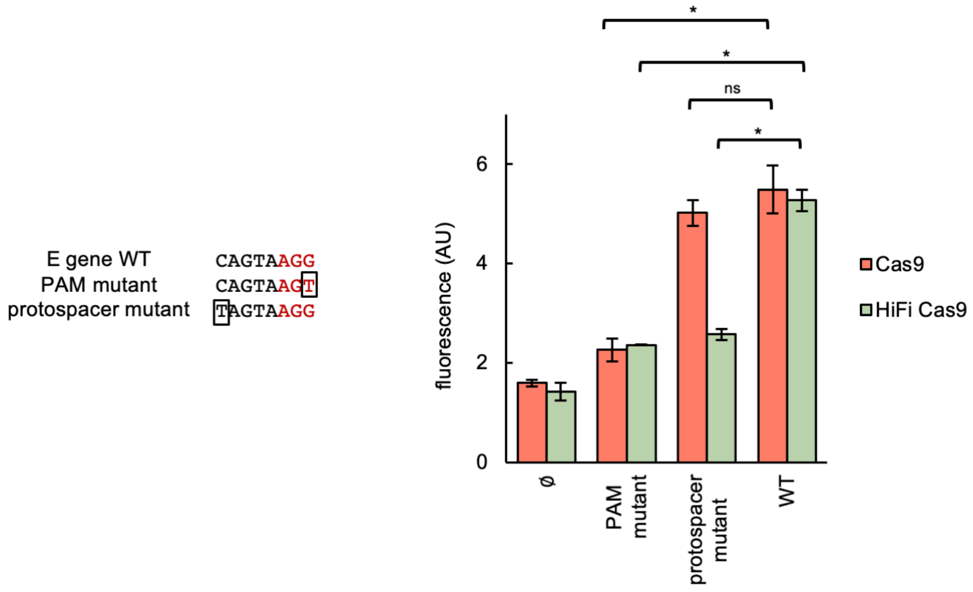

Figure S8: Detection of substitution mutations through CRISPR-Cas9-based strand displacement. Fluorescence-based characterization of E gene variant detection with the native Cas9 and HiFi Cas9. On the left, substitution mutations are framed (PAM shown in red). Error bars correspond to standard deviations (n = 3). *Statistical significance (Welch's t-test, two-tailed P < 0.05). ^{ns}Not statistically significant. These results show that the native Cas9 is only able to discriminate mutations in the PAM, while the HiFi Cas9 is able to discriminate mutations in the PAM and the protospacer, so this latter nuclease seems better suited for applications in which the detection of variants is required.

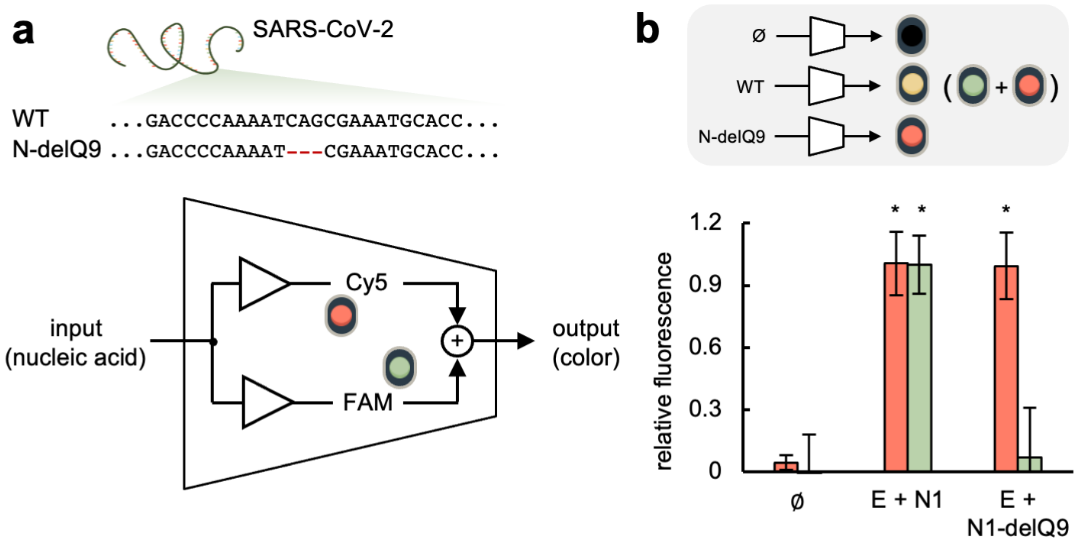

Figure S9: Mutant SARS-CoV-2 detection through CRISPR-Cas9-based strand displacement. a) Schematics of an electronic circuit implementing a molecular program for detection: if the sample is free of SARS-CoV-2, there is no light signal; if it contains the wild-type SARS-CoV-2, a "yellow" signal is obtained (merging the signals from Cy5 and FAM); if it contains a SARS-CoV-2 that carries the mutation N-delQ9, a red signal is obtained (only from Cy5). b) Fluorescence-based characterization of the detection by working directly with DNA amplicons: none, E and N1 (simulating the wild-type SARS-CoV-2), and E and N1-delQ9 (simulating a mutant SARS-CoV-2). Error bars correspond to standard deviations (n = 3). *Statistical significance (Welch's t-test, two-tailed $P < 0.05$).

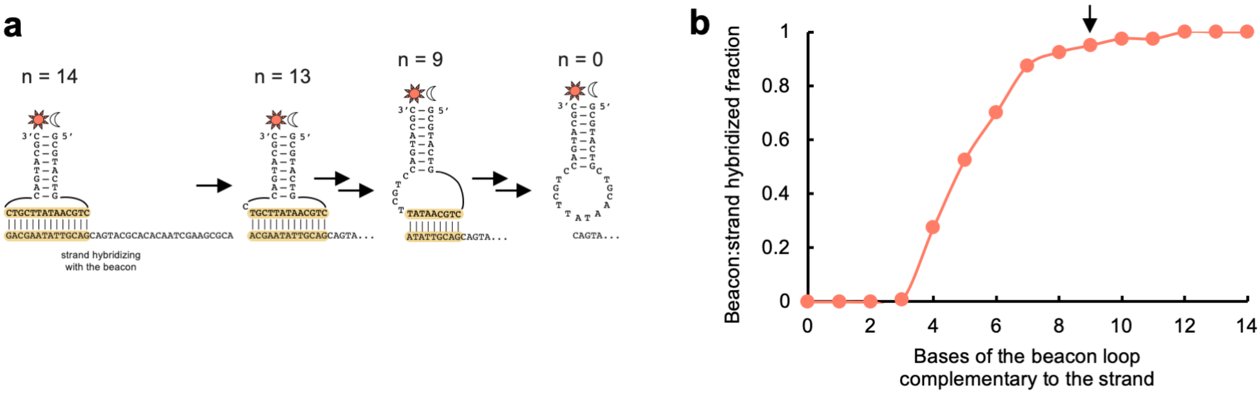

Figure S10: Computational analysis of the energy barrier associated with the interaction of the beacon. a) Schematics of the hybridization between a beacon and a given strand. The beacon for the SARS-CoV-2 E gene was considered (fixed). Different sequences (based on the E gene) were generated to interact with the beacon, starting from one that interacts with the full-length loop (seed region of 14 nt, n = 14) and ending

with one that does not interact with the loop (no seed region, n = 0). b) Molar fraction of the hybridized species (beacon:strand) as a function of the size of the seed region (*i.e.*, the number of bases of the beacon loop that are complementary to the strand). NUPACK was used considering DNA parameters and a concentration of 40 nM for both species. The arrow marks the sequence corresponding to the actual design.

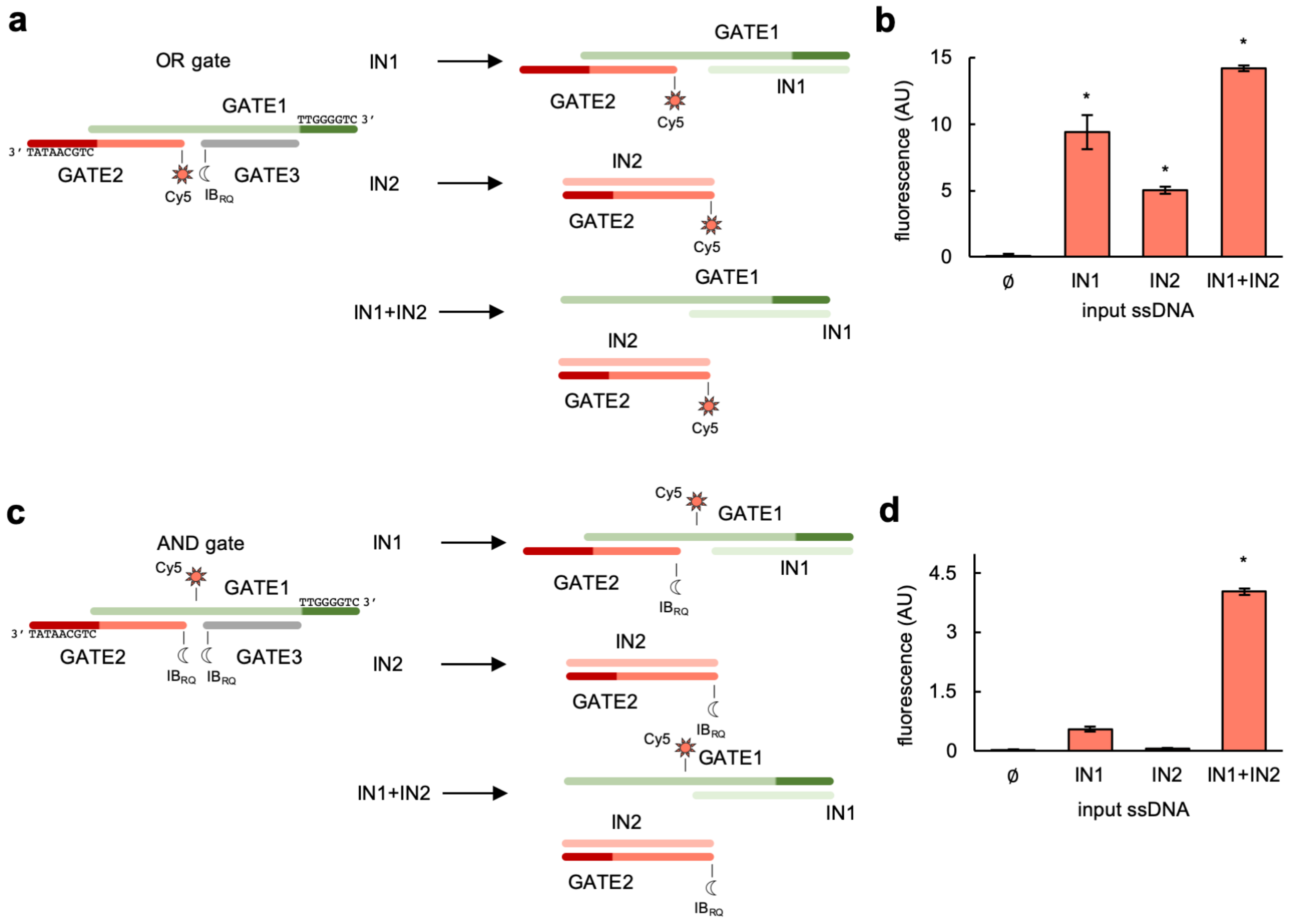

Figure S11: Characterization of the logic circuits with input ssDNA. a) Schematics of the OR gate in a pure DNA strand displacement scenario (the oligonucleotides IN1 and IN2 were the inputs). GATE1 was not labelled, GATE2 was labelled with the fluorophore Cy5 (sun icon) in the 5' end, and GATE3 was labelled with the dark quencher Iowa Black RQ (moon icon) in the 3' end. The sequences of the toeholds (seed regions) are shown. b) Fluorescence-based results for the OR gate. c) Schematics of the AND gate in a pure DNA strand displacement scenario. GATE1 was labelled with Cy5 internally, GATE2 was labelled with Iowa Black RQ in the 5' end, and GATE3 was labelled with Iowa Black RQ in the 3' end. d) Fluorescence-based results for the AND gate. Error bars correspond to standard deviations (n = 3). *Statistical significance (Welch's t-test, two-tailed $P < 0.05$).

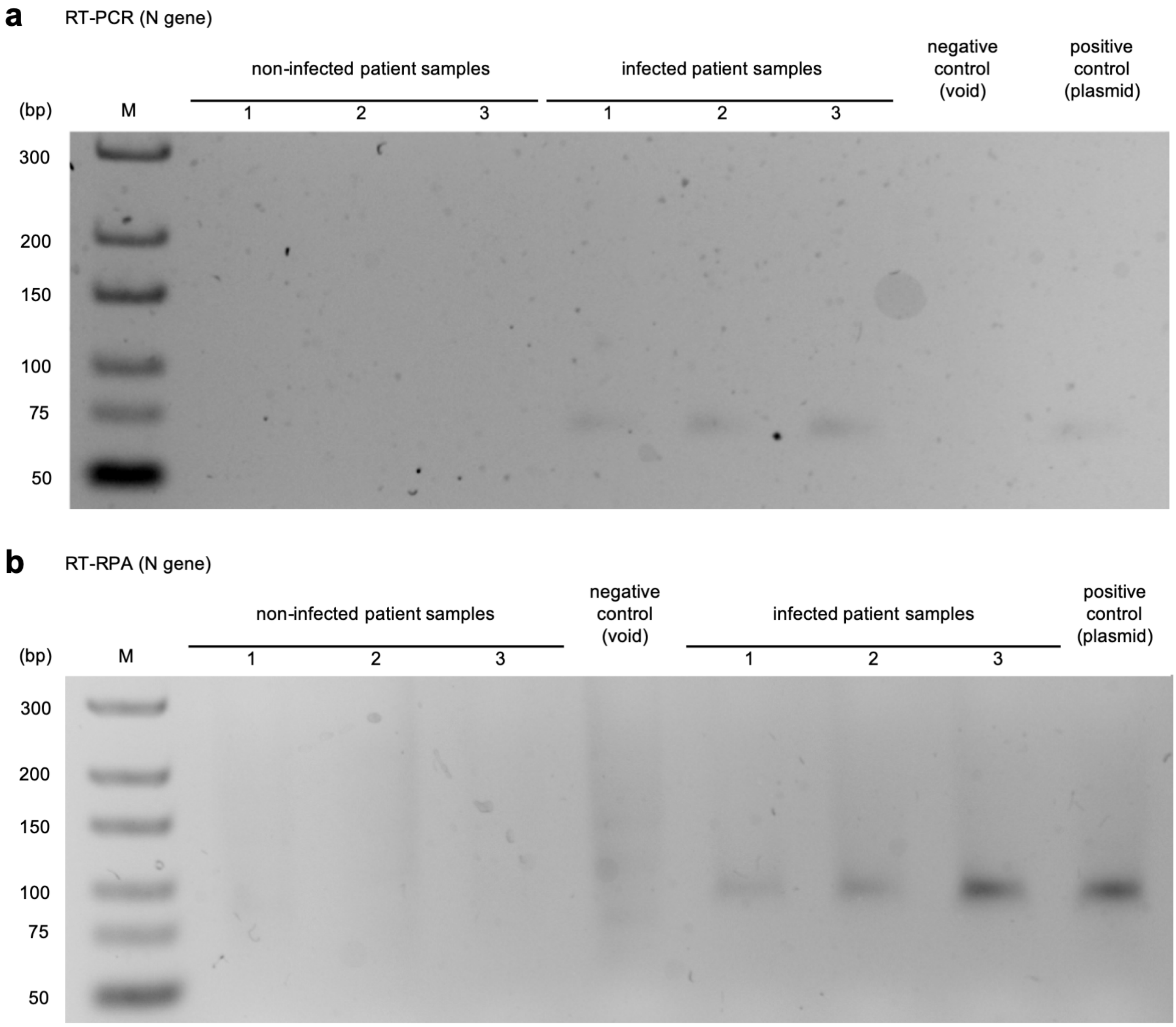

Figure S12: Gel electrophoretic assay to reveal the viral genome amplification. a) Amplification of SARS-CoV-2 (N gene) over patient samples by RT-PCR. b) Amplification of SARS-CoV-2 (N gene) over patient samples by RT-RPA. Due to the RPA buffer composition, we noted that the migration of the fragment in the gel was slower. M, molecular marker (GeneRuler ultra-low range DNA ladder).
